# Comments on Triassi *et al.* Environmental Pollution from Illegal Waste Disposal and Health Effects: A Review on the “Triangle of Death”. *Int. J. Environ. Res. Public Health* 2015, *12*, 1216–1236

**DOI:** 10.3390/ijerph120303358

**Published:** 2015-03-19

**Authors:** Maurizio Bifulco

**Affiliations:** Faculty of Pharmacy and Medicine, University of Salerno, Via Salvatore Allende, 84081 Baronissi Salerno, Italy; E-Mail: maubiful@unisa.it; Tel.: +39-089-96-9742; Fax: +39-089-96-9602

A recent paper was published on *Int. J. Environ. Res. Public Health* addressing the so-called “Triangle of Death” linked to waste crisis in the Southern Italy [1]. Three decades of illegal waste management including uncontrolled disposal of toxic and industrial material, land filling and unauthorized incineration have transformed these Italian areas, into the poisoned dustbin of the country, the “Triangle of Death” also called “Land of Fires” [2,3], characterized by the presence of a widespread organized crime, a huge social and economic disintegration, a high population density with large migration flows. This phenomenon arose a dangerous and uncontrollable alarmism in the population with unpredictable socio-economic damages. In my opinion, despite preliminary scientific studies suggest a link between hazardous exposure from toxic wastes and cancer occurrence [4], to definitively clarify this point further extensive and analytical studies are required. In particular, an accurate mapping and characterization of all the potentially contaminated sites and groundwaters, as well as agricultural lands subjected to illegal spills, need to be urgently developed, together with a more detailed epidemiological investigation aimed to explore the impact of illegal pollutants on environmental matrices (soil, water, air), on the food chain and, especially, on human health. The improvement of scientific knowledge and its translational relevance on environment and health concerns represents, indeed, the unique valuable tool to understand the critical elements of contaminated areas, to give evidence for a public claim and, as a consequence, to implement preventive measures and precautions in the Land of Fires. Moreover, analytical methods based on exposomic approach, as well as human biomonitoring programs and molecular genomic instruments, allow a broad and dynamic view of individual environmental exposure to complex mixtures of contaminants during the whole life cycle, the so-called enviroma, which unquestionably contributes, together with genetic factors, to the environment-related disease pathogenesis. According to the experimental procedure, in order to deepen the potential association between environment and common diseases in contaminated areas, the detection of personal exposure, obtained through filling out questionnaires about individual habits and the use of the latest generation equipment (sensors connected devices of exposure, remote sensing instruments, and smartphones) is associated with the comparative assessment of selected biomarkers, like individual gene-expression, protein, and metabolic profile time variation. For this reason, the Land of Fires could constitute an ideal open-air laboratory for a promising exposomic research, in order to characterize pollution types and sources and to elaborate measures to eliminate or reduce associated health risks [4]. The approval of prevention interventions, according to an established European model, should pass through a rigorous Health Impact Assessment (HIA), based on scientific evidence and on integration of several technical expertise, achieving maximum health benefits, and minimizing adverse effects. This is an ambitious project proposal, which certainly requires massive public and private funding. Furthermore, an adequate information system could directly support and encourage public participation and institutional activities in the environmental surveillance and monitoring in order to guide with success, on the basis of the precautionary principle, the rapid implementations of preventive measures allowing to make decisive actions for the Campania emergency.
